# Background factors involved in the epidemiology of functional constipation in the Japanese population: a cross-sectional study

**DOI:** 10.1186/s13030-022-00237-2

**Published:** 2022-03-10

**Authors:** Sayuri Yamamoto, Wataru Ohashi, Yoshiharu Yamaguchi, Shunsuke Inamoto, Akira Koshino, Tomoya Sugiyama, Kazuhiro Nagao, Yasuhiro Tamura, Shinya Izawa, Masahide Ebi, Jun Usami, Koichi Hamano, Junko Izumi, Yoshinori Wakita, Yasushi Funaki, Naotaka Ogasawara, Makoto Sasaki, Masato Maekawa, Kunio Kasugai

**Affiliations:** 1 Division of Gastroenterology, Aichi Medical University, 1-1 Yazakokarimata, Nagakute, Aichi Prefecture 480-1195 Japan; 2 Division of General Medicine, Aichi Medical University, 1-1 Yazakokarimata, Nagakute, Aichi Prefecture 480-1195 Japan; 3 Division of Biostatistics, Clinical Research Center, Aichi Medical University, Nagakute, Aichi Prefecture Japan

**Keywords:** Functional constipation, Population composition ratio, Online survey, Awareness of constipation, Background factors

## Abstract

**Background:**

Functional constipation (FC), a functional bowel disorder with symptoms of constipation, has considerable impact on quality of life. As data regarding its prevalence and epidemiology are lacking, this study aimed to evaluate the prevalence, population composition, lifestyle, quality of life, and clinical characteristics of these individuals by comparing people with and without FC. These parameters were also compared among individuals with strong and weak awareness of constipation.

**Methods:**

An internet survey was conducted among 10,000 individuals aged 20–69 years from the general Japanese population; they were registered with an internet survey company. The following data were obtained: age, sex, educational history, occupation, residence, history of other diseases, lifestyle (including smoking/drinking habits using the Japanese Health Practice Index, medication use, symptoms of constipation according to the Rome III criteria, stool types according to the Bristol stool scale, and use of laxatives, including the place of purchase and cost per month or acceptable cost per month. The 8-item Short Form Health Survey Questionnaire was also used; FC was diagnosed based on Rome III criteria. All respondents were classified according to their awareness of constipation (i.e. strong or weak), and their characteristic features were compared.

**Results:**

The data of 3000 respondents were evaluated; 262 (8.7%) had FC, which was common among older adults, women, and homemakers. FC was associated with changes in the frequency of bowel movement, sensation of incomplete or scanty evacuation, and the use of manual maneuvers; these are consequential clinical symptoms of FC. These individuals frequently skipped breakfast, had insufficient sleep, had more severe constipation, and had purchased laxatives in pharmacies or online more often than those without FC. A strong awareness of constipation was significantly more prevalent among women and homemakers. A history of anemia and cardiovascular disease was significantly more frequent in the strong awareness group, whereas a history of hypertension was more frequent in the weak awareness group.

**Conclusions:**

Appropriate and comprehensive management should be provided for FC, based on the understanding of its characteristic features and considering the symptoms and lifestyle.

## Background

The definition and diagnostic criteria for chronic constipation have been recently updated in the Rome IV criteria [1]. According to this classification, functional constipation (FC) is diagnosed when two or more of the following six symptoms are present: “straining during at least 25% of defecations”, “lumpy or hard stools (type 6 or 7 on the Bristol stool scale)” [2, 3], “sensation of incomplete evacuation,” “sensation of anorectal obstruction,” “manual maneuvers to facilitate defecation,” and “fewer than three defecations per week”. It is also diagnosed when both the following conditions are met: “loose stools rarely present without use of laxatives” and “does not meet Rome IV criteria for irritable bowel syndrome (IBS)”. For defining chronic constipation, these conditions must be met for the past 3 months, with symptom onset at least 6 months prior to diagnosis [4]. The Japanese Clinical Guidelines for Chronic Constipation 2017 defined constipation as a “state in which feces that should be eliminated from the body cannot be passed in sufficient quantity and in comfort”. The guidelines explained that it can “cause symptoms requiring examination and treatment due to reduced frequency of defecation (e.g. abdominal pain and abdominal bloating, among others), hard stools (e.g. difficulty in defecating and excessive straining during defecation, among others), or evacuation disorder (e.g. difficulty in defecating even loose stools, excessive straining during defecation, sensation of incomplete evacuation and therefore frequent defecation).” Reports suggest that chronic constipation affects 1–27% of the population, with the wide range attributed to variations in the population studied, definition used, and evaluation methods [4]. Chronic constipation is a highly prevalent disorder, characterized by gastrointestinal symptoms. It is frequently encountered in different medical specialties, and not only in gastroenterology [5]. A study on the impact of various functional gastrointestinal disorders, including constipation, on survival reported that chronic constipation conferred a significantly higher risk of poorer survival than other disorders, and it should therefore be given due consideration [6]. Prior to the establishment of guidelines, initial diagnoses and treatment approaches were based on the individual experience of the physician. Thus, there is limited information on the prevalence and actual disease status of patients with constipation. Moreover, few patients seek consultation for constipation as their chief complaint. Those with the condition either tend to self-medicate using over-the-counter (OTC) medications, or modify their lifestyle themselves [5, 7–9]. This is due to the fact that constipation is not regarded as a disease [10], there is no clear definition or diagnostic criteria for chronic constipation, and initial diagnosis and treatment approaches are based on clinical experience. Although evidence-based management of constipation has recently been promoted since the publication of the Clinical Guidelines for Chronic Constipation 2017 [4], there are only a few reports of its large-scale implementation [8, 11, 12]. In Japan, there are only two existing reports [8, 13] on the public awareness of constipation, its actual incidence, and on medication use and quality of life (QOL) of these patients. Therefore, much remains unknown regarding the epidemiology of FC and its background, including the status of constipation, medication, and treatment satisfaction. In this study, we performed an online survey to determine the actual prevalence of FC in Japan to investigate the frequency of symptoms, background factors, treatment, and QOL.

In Japanese subjects with awareness of constipation, the frequency and FC-related factors as per the Rome III criteria [14] were compared to those of individuals without FC (non-FC). Factors associated with a strong awareness of constipation were also studied.

## Methods

### Subjects

The survey was conducted between October 8 and 11, 2016, among 10,000 individuals aged 20–69 years from the Japanese general population. They were registered with Rakuten Insight (Osaka, Japan), an internet survey company. All survey participants provided informed consent. Valid answers were received from 9523 subjects, 4908 (51.5%) who indicated “I strongly think I have constipation” or “I think I have constipation” in response to the question, “Do you think you have constipation?” In this context, the awareness of constipation is a subjective sensation that can depend on the individual’s strength of awareness. The perceived strength of the condition may be affected by differing lifestyles and background circumstances. In this study, we used a highly subjective index of the strength of constipation awareness to evaluate real-world ordinary consumers based on patient reported outcomes. This was achieved using only the above question. Among the 4908 subjects with awareness of constipation, 3000 were randomly extracted by fitting the general population composition ratio based on prefecture, sex, and age. This was considered to reflect the demographic profile in Japan, as estimated by the Statistics Bureau, Ministry of Internal Affairs and Communications of Japan as of October 1, 2014.

### Exclusion criteria

Those with organic diseases such as cancer and inflammatory diseases, neurological or endocrine disorders, and secondary constipation induced by medication (e.g. opioids, antidepressants, anticholinergic agents, calcium blockers, and proton pump inhibitors, among others) were excluded from the survey.

### Survey

The information obtained from each study participant included the following: age, sex, educational history, occupation, residence, history of other diseases, lifestyle (including smoking/drinking habits using the Japanese Health Practice Index [JHPI]), medication use, symptoms of constipation according to the Rome III criteria [14], stool types according to the Bristol stool scale [2, 3], and use of laxatives (including the place of purchase and cost per month or acceptable cost per month). Participants also responded to an 8-item Short Form Health Survey Questionnaire (SF-8) [15]. FC was diagnosed based on the Rome III criteria [14], which differs from the Rome IV criteria in terms of the diagnosis of IBS. As per the former, the diagnosis of IBS entails chronic abdominal pain or discomfort experienced for at least 3 days per month instead of at least 1 day per week.

Among the 3000 participants whose data were analyzed, those who responded “I think so” to the question “Do you usually think that you have constipation?” were classified into the strong awareness group, and the rest were classified into the weak awareness group. A comparative study was conducted among the groups.

### Statistical analysis

The level of significance for the statistical analysis was set at *P* < 0.05 (two-sided), and the two-sided 95% confidence interval was calculated where appropriate. To calculate the confidence interval of a proportion and the 95% confidence interval of a proportion of cases, the exact method (Clopper-Pearson) based on the F-distribution was used. Unpaired *t*-tests were used to compare continuous data between the two groups. We used the Fisher’s exact test to compare categorical (nominal) variables and investigate whether the proportions of one variable differed from the values of another. In addition, we used pairwise comparisons between proportions, with Benjamini-Hochberg adjustment of the *P* values, to further identify the categories of nominal variables showing significant differences. Logistic regression, with awareness of constipation as a dependent variable, was used to analyze factors related to awareness of constipation. For multivariate analysis, all variables were entered using forced entry. The Cochran-Armitage trend test was used to evaluate stool form by cost per month or acceptable cost per month.

### Ethical approval

This study was approved by the Institutional Review Board of the Aichi Medical University (approval number: 2016-M025; approved on October 6, 2016). It was performed in compliance with the principles of the Declaration of Helsinki, and the Ethical Guidelines for Medical and Health Research Involving Human Subjects, enacted by the Japanese Ministry of Education, Culture, Sports, Science and Technology and the Ministry of Health, Labour and Welfare (December 22, 2014). The internet survey and statistical analysis of the data were outsourced to Rakuten Insight, Inc. (Osaka, Japan). Participants were free to withdraw their consent at any time via the Internet.

## Results

### Comparison of functional and non-functional constipation in the Japanese population

#### Background factors

**Fig. 1:**
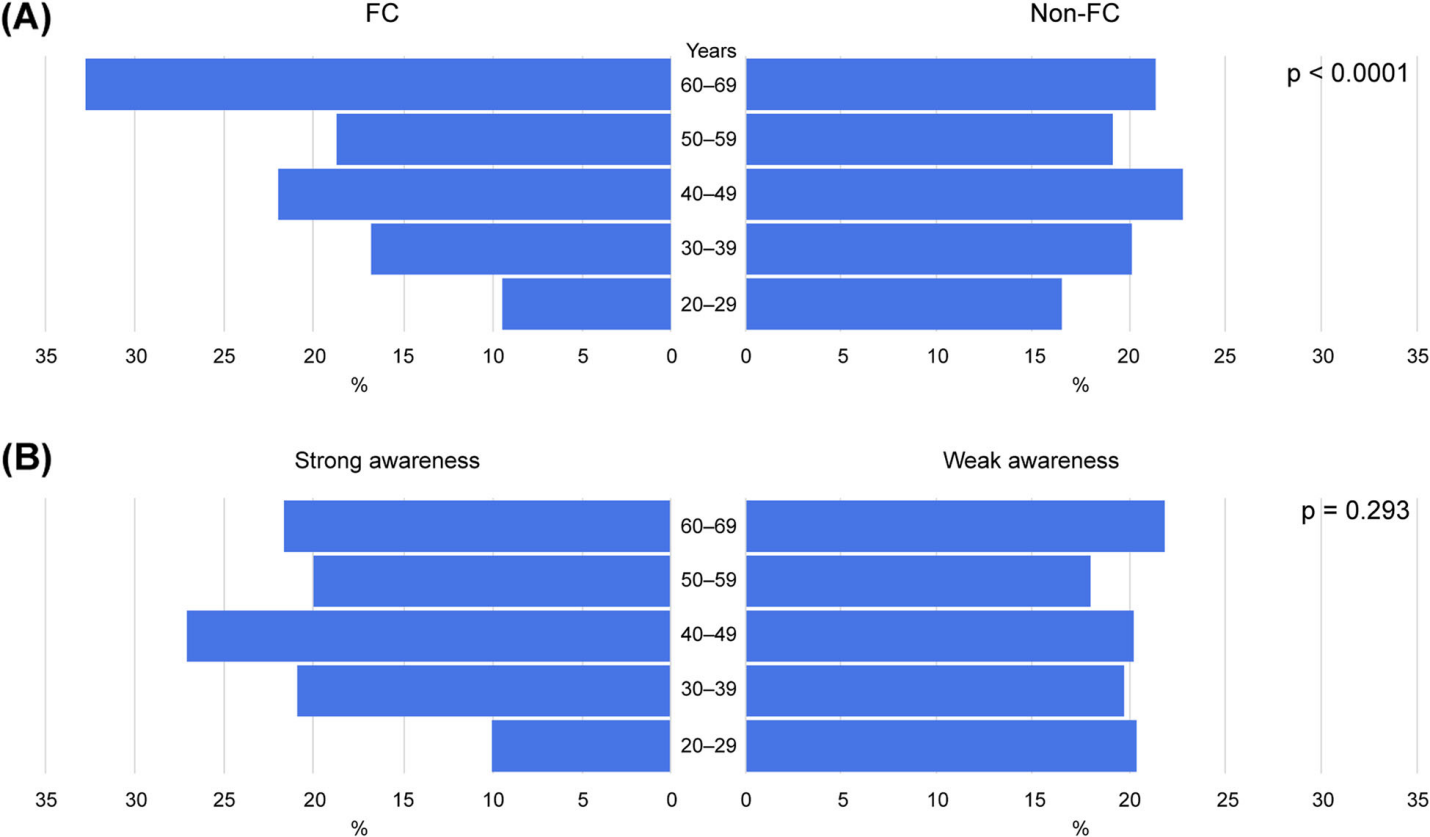
Age trends. Comparison of age range between FC and non-FC groups (**A**) and between groups with strong and weak awareness of constipation (**B**)

Among the 3000 subjects included, 262 (8.7%) and 2738 (91.3%) were classified into the FC and non-FC groups, respectively (Table 1). The percentage of women was significantly higher in the FC than in the non-FC group (72.1 and 47.8%, respectively; *P* < 0.001). Similarly, the mean age was significantly higher in the FC group (49.8 ± 13.1 vs. 45.8 ± 13.3 years; *P* < 0.001). The demographic trend showed a higher occurrence of FC in older adults, with a significantly higher frequency in those aged 60 and older (Fig. 1A). The non-FC group had a significantly higher body mass index (BMI) than the FC group (21.7 ± 3.6 vs. 21.0 ± 3.3 kg/m^2^, respectively; *P* = 0.002); however, the BMI in both groups was lower than the national average.

**Table 1:** Univariate analysis of participant characteristics in the functional constipation (FC) and non-FC groups, presented as the frequency or mean ± standard deviation (SD)

Items		FC (*n* = 262)	Non-FC (*n* = 2738)	*P* value*
Residential area: North (Tohoku, Hokkaido)/East (Kanto, Tokai, Koshinetsu)/West (Kinki, Chugoku, Shikoku, Kyushu), n/n/n		33/128/101	298/1483/959	0.250
Age, years (mean ± SD)		49.8 ± 13.1	45.8 ± 13.3	< 0.001**
Sex, female, n (%)		189 (72.1)	1308 (47.8)	< 0.001**
BMI, kg/m^2^ (mean ± SD)		21.0 ± 3.3	21.7 ± 3.6	0.002**
Jobs and education				
Employment status, n (%)	Student	10 (3.8%)	34 (1.2%)	0.360
	Office worker	103 (39.3%)	1266 (46.2%)	0.030**
	Self-employed	21 (8.0%)	216 (7.9%)	0.900
	Part-time worker	36 (13.7%)	389 (14.2%)	0.930
	Retired or unemployed	29 (11.1%)	439 (16.0%)	0.030**
	Homemaker	72 (27.5%)	394 (14.4%)	< 0.001**
Education, n (%)	Junior high school or high school	129 (49.2%)	1218 (44.5%)	0.150
	Bachelor’s degree or higher	132 (50.4%)	1506 (55.0%)	0.150
Past history of disease				
Hypertension, n (%)		25 (9.5%)	377 (13.8%)	0.060
Type 2 diabetes mellitus, n (%)		3 (1.1%)	196 (7.2%)	< 0.001**
Hyperlipidemia, n (%)		25 (9.5%)	313 (11.4%)	0.410
Lesion in the stomach, duodenum or small intestine, n (%)		0	291 (10.6%)	NA
Inflammatory bowel disease, n (%)		0	0	–
Hemorrhoids, n (%)		0	395 (14.4%)	NA
Diverticulum, n (%)		0	35 (1.3%)	NA
Gastrointestinal cancer, n (%)		0	0	–
Cerebrovascular disease, n (%)		0	60 (2.2%)	NA
Chronic obstructive pulmonary disease, n (%)		0	5 (0.2%)	NA
Liver disease, n (%)		0	62 (2.3%)	NA
Kidney disease, n (%)		0	62 (2.3%)	NA
Abdominal surgery without appendectomy, n (%)		0	0	–
Anemia, n (%)		36 (13.7%)	468 (17.1%)	0.190
Past treatment history				
Hypertensive drugs, n (%)		14 (5.3%)	276 (10.1%)	0.010**
Insulin injections or Hyperglycemia drugs, n (%)		0	123 (4.5%)	NA
Hyperlipidemia drugs, n (%)		22 (8.4%)	264 (9.6%)	0.580
Cerebrovascular disease, n (%)		0	80 (2.9%)	NA
Cardiovascular disease, n (%)		1 (0.4%)	81 (3.0%)	0.010**
Chronic renal failure or history of dialysis, n (%)		0	26 (0.9%)	NA
Depression or anxiety, n (%)		0	314 (11.5%)	NA
Lifestyle factors based on JHPI questionnaire				
Smoking more than 100 cigarettes per month and smoking for 6 month or longer, n (%)		41 (15.6%)	39 (1.4%)	0.580
Alcohol drinking occasionally or daily, n (%)		156 (59.5%)	1046 (38.2%)	0.900
Walking for 1 h/d, n (%)		100 (38.2%)	1027 (37.5%)	0.730
Body weight gain of at least 10 kg, n (%)		45 (17.2%)	630 (23.0%)	0.036**
Exercising for more than 30 min twice a week for at least 1 year, n (%)		57 (21.8%)	564 (20.6%)	0.630
Walking or similar exercise for more than 1 h/d, n (%)		56 (21.4%)	567 (20.7%)	0.610
Walking faster than other people of the same age, n (%)		97 (37.0%)	1038 (37.9%)	0.790
Body weight gain or loss of at least 3 kg within 1 year, n (%)		86 (32.8%)	928 (33.9%)	0.780
Eating faster than other people, n (%)		100 (38.2%)	1027 (37.5%)	0.730
Having dinner within 2 h before going to sleep at least 3 times a week, n (%)		67 (25.6%)	794 (29.0%)	0.290
Eating snacks after dinner at least 3 times a week, n (%)		83 (31.7%)	797 (29.1%)	0.390
Skipping breakfast at least 3 times a week, n (%)		69 (26.3%)	715 (26.1%)	0.940
Insufficient sleep, n (%)		144 (55.0%)	1588 (58.0%)	0.350
Strong awareness of constipation, n (%)		71 (27.1%)	1171 (42.8%)	< 0.001**
Rome III criteria question items				
Improvement with defecation, n (%)		194 (74.0%)	1933 (70.6%)	0.255
Onset associated with a change in frequency of stool, n (%)		133 (50.8%)	1613 (58.9%)	0.013**
Onset associated with a change in form (appearance) of stool, n (%)		137 (52.3%)	1577 (57.6%)	0.103
Straining during at least 25% of defecation, n (%)		211 (80.5%)	1828 (66.8%)	< 0.001**
Lumpy or hard stool at least 25% of defecations, n (%)		210 (80.2%)	1682 (61.4%)	< 0.001**
Sensation of incomplete evacuation for at least 25% of defecation, n (%)		230 (87.8%)	1828 (66.8%)	< 0.001**
Sensation of hard evacuation, n (%)		255 (97.3%)	2036 (74.4%)	< 0.001**
Manual maneuvers to facilitate digital evacuation at least 25% of defecation, n (%)		18 (6.9%)	144 (5.3%)	0.254
Manual maneuvers to facilitate support of the pelvic floor at least 25% of defecation, n (%)		62 (23.7%)	321 (11.7%)	< 0.001**
Loose stools are rarely present without the use of laxatives, n (%)		262 (100.0%)	843 (30.8%)	< 0.001**
Quality of life (SF-8)				
GH^†^ (mean ± SD)		46.9 ± 7.5	46.1 ± 7.8	0.121
PF^‡^ (mean ± SD)		47.2 ± 9.5	46.9 ± 9.0	0.532
RP^§^ (mean ± SD)		48.6 ± 7.1	47.9 ± 7.9	0.158
BP^¶^ (mean ± SD)		48.5 ± 8.3	48.3 ± 8.6	0.655
VT^††^ (mean ± SD)		48.1 ± 7.4	46.5 ± 7.9	0.002**
SF^‡‡^ (mean ± SD)		47.9 ± 9.1	45.9 ± 10.2	0.002**
MH^§§^ (mean ± SD)		48.9 ± 7.7	47.2 ± 8.3	0.001**
RE^¶¶^ (mean ± SD)		45.0 ± 10.5	43.1 ± 10.9	0.010**
PCS^†††^ (mean ± SD)		46.9 ± 7.1	46.9 ± 7.5	0.966
MCS^‡‡‡^ (mean ± SD)		46.7 ± 8.3	44.4 ± 9.2	< 0.001**

*The level of significance is set at *P* < 0.05

**Significant difference between groups

^†^*GH* general health, ^‡^*PF* physical functioning, ^§^*RP* role physical, ^¶^*BP* body pain, ^††^*VT* vitality, ^‡‡^*SF* social functioning, ^§§^*MH* mental health, ^¶¶^*RE* role emotional, ^†††^*PCS* physical component summary, ^‡‡‡^*MCS* mental component summary

A higher proportion of subjects in the non-FC group indicated that they were either office workers (46.2% vs. 39.3%, respectively; *P* = 0.030) or retired/unemployed (16.0% vs. 11.1%; *P* = 0.030). Conversely, there were significantly more homemakers in the FC group than in the non-FC group (27.5% vs. 14.4%, respectively; *P* < 0.001). Finally, the non-FC group had a higher proportion of individuals with a history of type 2 diabetes mellitus (7.2% vs. 1.1%; *P* < 0.001), hypertension (13.8% vs. 9.5%; *P* = 0.060), anemia (17.1% vs. 13.7%; *P* = 0.190), and hyperlipidemia (11.4% vs. 9.5%; *P* = 0.410), as well as a past treatment history of hypertension (10.1% vs. 5.3%; *P* = 0.010) and cardiovascular disease (3.0% vs. 0.4%; *P* = 0.010). However, significant differences were only noted in the history of type 2 diabetes mellitus and in the past treatment history of hypertension and cardiovascular disease.

#### Lifestyle

The JHPI lifestyle survey revealed that the occurrence of previous weight gain ≥10 kg was significantly more frequent in the non-FC group than in the FC group (23.0% vs. 17.2%; *P* = 0.036). No association was found between FC and drinking, smoking, eating, walking, or exercising. A strong awareness of constipation was a significant factor for not having FC (Table 1).

#### Quality of life

Evaluation of the QOL of participants using the SF-8 questionnaire [15] revealed that subjects in the FC group had a significantly higher mental component summary (MCS) than those in the non-FC group (46.7 ± 8.3 vs. 44.4 ± 9.2, respectively; *P* < 0.001); this included vitality (VT, feeling exhausted), social functioning (SF, having problems with family or friends), role emotional (RE, having difficulty in work or daily activity for psychological reasons), and mental health (MH, being nervous or depressed) (Table 1).

#### Clinical symptoms

**Fig. 2:**
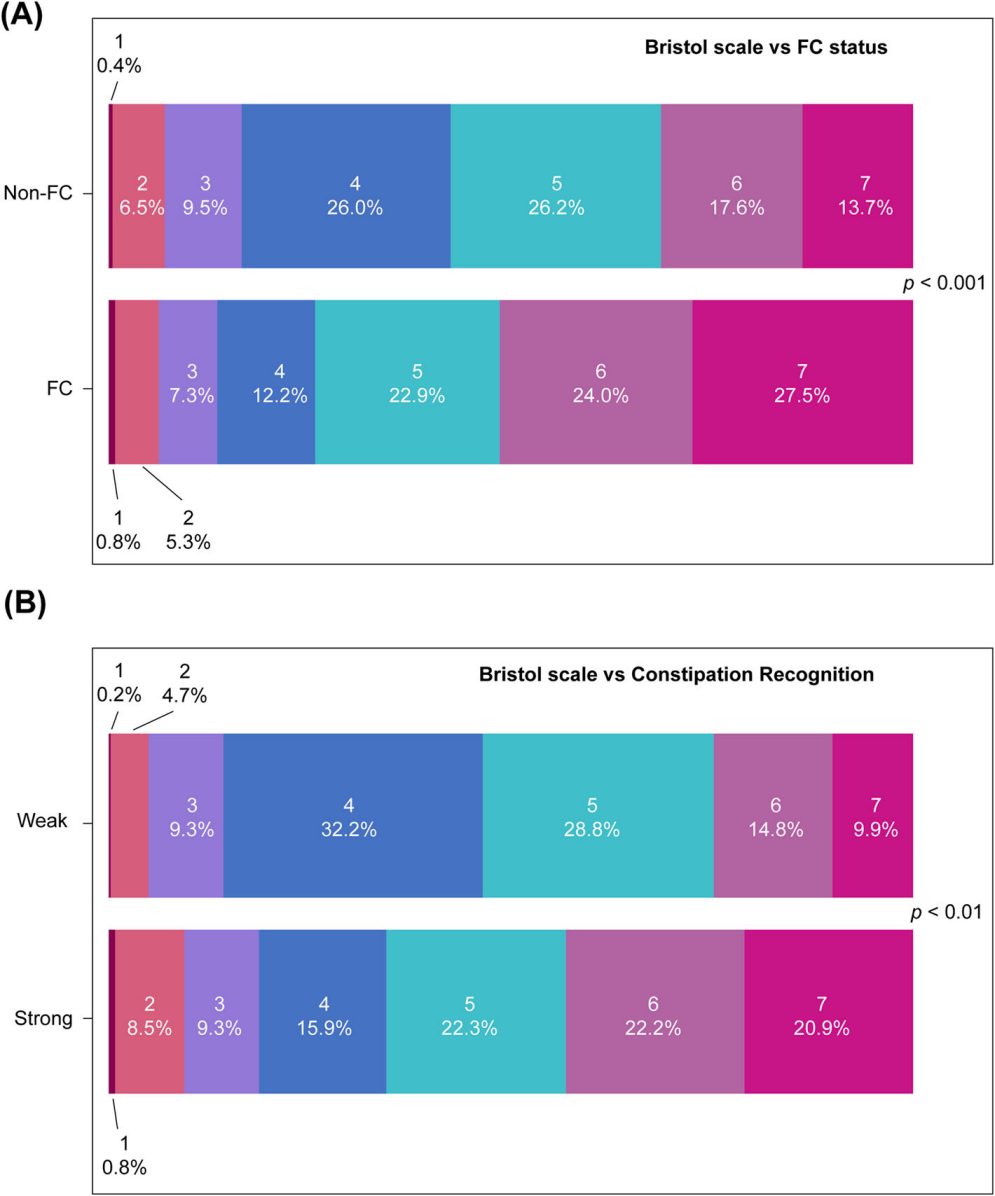
Bristol scale. Comparison of stool types between FC and non-FC groups (**A**) and between groups with strong and weak awareness of constipation (**B**). 1: Watery, no solid pieces; 2: fluffy pieces with ragged edges, a mushy stool; 3: soft blobs with clear cut, smooth and soft, passed early; 4: like sausage or snake, smooth and soft; 5: like sausage but with cracks on surface; 6: sausage shaped but lumpy; 7: shaped hard lumps, like nuts, hard to pass

A comparison of the two groups based on the Rome III criteria revealed that the following conditions occurred significantly more frequently in subjects in the FC group than in the non-FC group: straining, hard stool, sensation of incomplete evacuation, sensation of anorectal obstruction, rare bowel movements without the use of laxatives, and manual maneuvers to facilitate support of the pelvic floor at least 25% of defecation (Table 1). Loose stools that are rarely present without the use of laxatives is a Rome III criterion for FC. All FC subjects (100%) affirmed this symptom compared with only 30.8% of non-FC subjects (*P* < 0.001). Although there was no significant difference between the groups in terms of the use of manual maneuvers (6.9% vs. 5.3% in the FC and non-FC groups, respectively; *P* = 0.254), very few used manual maneuvers to facilitate defecation. A significantly lower percentage of subjects with FC had stools corresponding to Bristol stool scale type 4 (i.e. normal stool) compared with those without FC (12.2% vs. 26%, respectively; *P* < 0.001) (Fig. 2).

#### Source of laxative and acceptable cost

**Fig. 3:**
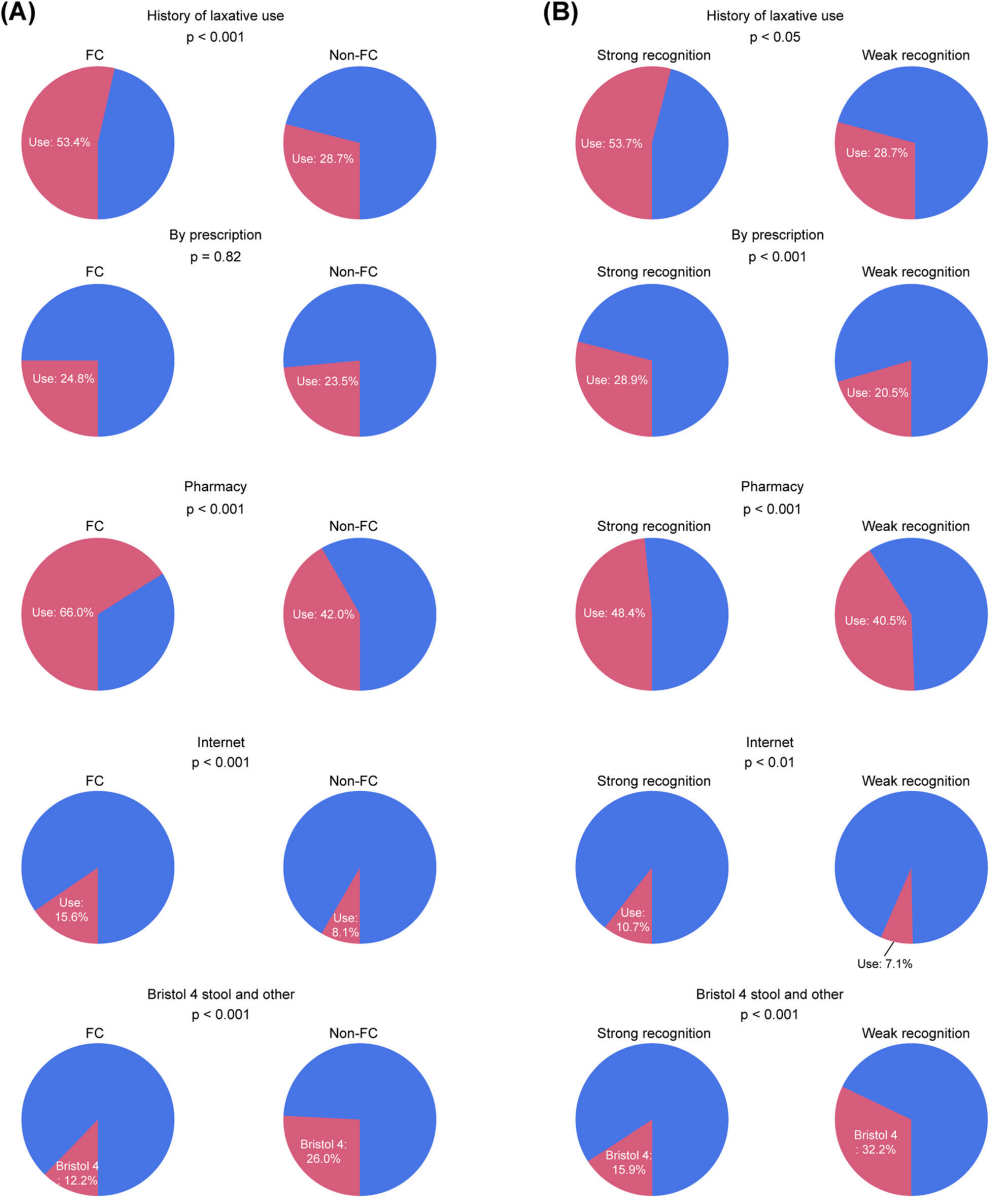
History of laxative use. Comparison of laxative purchase. Comparison of Bristol 4 stool scale between functional constipation (FC) and non-FC groups (**A**), and between groups with strong and weak awareness of constipation (**B**)

**Fig. 4:**
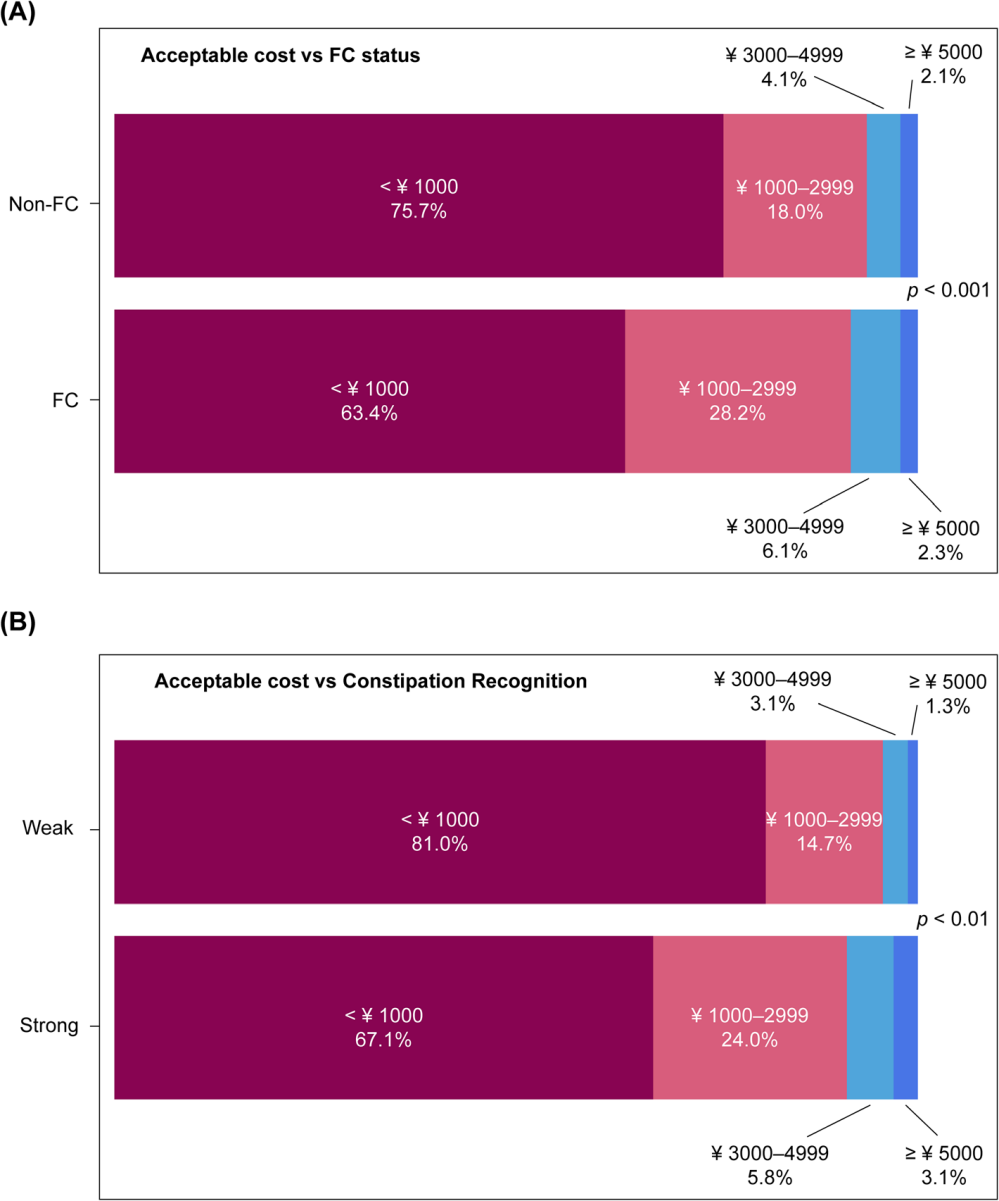
Acceptable cost of laxatives. Comparison of laxative costs between FC and non-FC groups (**A**) and between groups with strong and weak awareness of constipation (**B**)

A significantly higher proportion of FC subjects used laxatives compared with non-FC subjects (53.4% vs. 28.7%; *P* < 0.001). While no significant difference was found between the groups based on laxative purchase by physician’s prescription, laxative purchase in pharmacies, and laxative purchase online, these were more common in the FC group (Fig. 3). There was significantly more variation among FC subjects in terms of the amount they were willing to pay for laxatives (*P* < 0.001), and they paid a significantly higher amount than non-FC subjects (*P* < 0.001) (Fig. 4).

#### Significant background factors

Factors found to be significant on univariate analysis in the FC group were included in the multivariate logistic regression analysis, using a detection rate of ≤0.2%. Age, sex, and certain clinical symptoms based on the Rome III criteria, including sensation of incomplete evacuation for at least 25% of defecation, sensation of hard evacuation, and manual maneuvers for facilitating support of the pelvic floor for at least 25% of defecation, were found to be significant background factors related to FC. Factors found to be significant for non-FC were onset associated with a change in frequency of stool, and Bristol stool type 4. (Table 2).

**Table 2:** Multivariate analysis of significant background factors for functional constipation (FC)

Items	Univariate				Multivariate			
	Odds	95% CI		***P*** value*	Odds	95% CI		***P*** value*
Age	1.023	1.013	1.033	< 0.001**	1.032	1.020	1.045	< 0.001**
Sex (female = 1)	0.353	0.267	0.468	< 0.001**	0.380	0.260	0.498	< 0.001**
BMI	0.942	0.906	0.980	0.003**				
Jobs and education								
Office worker	0.753	0.581	0.976	0.032**				
Retired or unemployed	0.677	0.449	1.023	0.064				
Homemaker	2.255	1.685	3.018	< 0.001**				
Bachelor’s degree or higher	0.634	0.485	0.827	< 0.001**				
Past history of disease								
Hypertension	0.629	0.414	0.956	0.030**				
Type 2 diabetes mellitus	0.150	0.048	0.473	0.001**	0.191	0.058	0.627	0.006**
Dyslipidemia	0.689	0.459	1.034	0.072				
Anemia	0.213	0.149	0.305	< 0.001**	0.173	0.119	0.252	< 0.001**
Rome III criteria question items								
Improvement with defecation	1.188	0.891	1.585	0.241				
Onset associated with a change in frequency of stool	0.719	0.558	0.927	0.011**	0.689	0.477	0.996	0.048**
Onset associated with a change in form or appearance of stool	0.807	0.626	1.040	0.098				
Straining during at least 25% of defecation	2.060	1.502	2.825	< 0.001**				
Lumpy or hard stools at least 25% of defecations	2.535	1.854	3.468	< 0.001**				
Sensation of incomplete evacuation for at least 25% of defecation	3.578	2.451	5.223	< 0.001**	1.953	1.283	2.974	0.002**
Sensation of hard evacuation	12.56	5.899	26.744	< 0.001**	8.152	3.600	1.846	< 0.001**
Manual maneuvers to facilitate digital evacuation for at least 25% of defecation	1.329	0.801	2.208	0.271				
Manual maneuvers to facilitate support of the pelvic floor for at least 25% of defecation	2.334	1.716	3.176	< 0.001**	1.587	1.123	2.241	0.009**
Loose stools are rarely present without the use of laxatives	–	–	–	–	–	–	–	–
Bristol stool type 4	0.395	0.270	0.578	< 0.001**	0.585	0.386	0.888	0.012**
Source of laxative								
History of laxative use	5.851	4.341	7.886	< 0.001**				
Pharmacy	2.684	2.056	3.505	< 0.001**				
Internet	2.103	1.446	3.015	< 0.001**				
Acceptable laxative cost								
More than 1000 yen	1.806	1.384	2.357	< 0.001**				

*The level of significance is set at *P* < 0.05

**Significant difference between groups

### Comparison between strong and weak awareness of constipation

#### Background factors

The survey participants (*n* = 3000) were classified into either strong awareness or weak awareness of constipation groups. Strong awareness was significantly more prevalent in women than in men (57.3% vs. 42.7%; *P* < 0.001) (Table 3); however, a stronger awareness of constipation was observed among men in their 40s (Fig. 1B). The average BMI was significantly higher in the weak awareness group; however, the subjects in both groups had a lower mean BMI than the national average in Japan.

**Table 3:** Univariate analysis of characteristics in participants with strong or weak awareness of constipation, presented as the frequency or mean ± standard deviation (SD)

Items		Strong awareness of constipation (***n*** = 1362)	Weak awareness of constipation (***n*** = 1638)	***P*** value*
Residential area	North (Tohoku, Hokkaido)/East (Kanto, Tokai, Koshinetsu)/West (Kinki, Chugoku, Shikoku, Kyushu), n/n/n	136/736/490	193/875/570	0.280
Age	Years, (mean ± SD)	46.3 ± 12.9	46.0 ± 13.8	0.500
Sex	Female, (%)	57.3	48.8	< 0.001**
BMI	kg/m^2^ (mean ± SD)	21.4 ± 3.6	21.8 ± 3.5	0.001**
Jobs and education		n	n	
Jobs, n (%)	Student	15 (1.1%)	20 (1.2%)	0.850
	Office worker	619 (45.4%)	750 (45.8%)	0.870
	Self-employed	108 (7.9%)	129 (7.9%)	> 0.999
	Part-time worker	191 (14.0%)	234 (14.3%)	0.870
	Retired or unemployed	190 (14.0%)	278 (17.0%)	0.030**
	Homemaker	239 (17.5%)	227 (13.9%)	0.006**
Education, n (%)	Junior high school, High school	647 (47.5%)	715 (43.7%)	0.010**
	Bachelor’s degree or over	712 (52.3%)	650 (39.7%)	0.020**
Past history of disease		n	n	
Hypertension, n (%)		161 (11.8%)	241 (14.7%)	0.020**
Type 2 diabetes mellitus, n (%)		90 (6.6%)	95 (5.8%)	0.360
Hyperlipidemia, n (%)		153 (11.2%)	185 (11.3%)	> 0.999
Lesion in the stomach, duodenum or small intestine, n (%)		131 (9.6%)	160 (9.8%)	0.900
Inflammatory bowel disease, n (%)		0	0	
Hemorrhoids, n (%)		161 (11.8%)	234 (14.3%)	0.051
Diverticulum, n (%)		11 (0.8%)	24 (1.5%)	0.120
Gastrointestinal cancer, n (%)		0	0	
Cerebrovascular disease, n (%)		32 (2.3%)	28 (1.7%)	0.240
Chronic obstructive pulmonary disease, n (%)		1 (0.1%)	4 (0.2%)	0.400
Liver disease, n (%)		27 (2.0%)	35 (2.1%)	0.800
Kidney disease, n (%)		31 (2.3%)	31 (1.9%)	0.520
Abdominal surgery without appendectomy, n (%)		0	0	
Anemia, n (%)		263 (19.3%)	241 (14.7%)	< 0.001**
Past treatment history				
Hypertensive drugs, n (%)		123 (9.0%)	167 (10.2%)	0.290
Insulin injections or hyperglycemia drugs, n (%)		59 (4.3%)	64 (3.9%)	0.580
Hyperlipidemia drugs, n (%)		145 (10.6%)	141 (8.6%)	0.060
Cerebrovascular disease, n (%)		43 (3.2%)	37 (2.3%)	0.140
Cardiovascular disease, n (%)		47 (3.5%)	35 (2.1%)	0.030**
Chronic renal failure or history of dialysis, n (%)		12 (0.9%)	14 (0.9%)	> 0.999
Depression or anxiety, n (%)		157 (11.5%)	157 (9.6%)	0.090
Lifestyle factors based on JHPI questionnaire				
Smoking more than 100 sticks per month, n (%)		197 (14.5%)	236 (14.4%)	0.920
Alcohol consumption, n (%)		804 (59.0%)	998 (60.9%)	0.310
Body weight increased by 10 kg or more from the age of 20, n (%)		315 (23.1%)	359 (21.9%)	0.430
Exercising for more than 30 min twice a week for at least 1 year		259 (19.0%)	360 (22.0%)	0.040**
Walking for 1 h/d, n (%)		520 (38.2%)	614 (37.5%)	0.730
Walking faster than other people of the same age, n (%)		463 (34.0%)	621 (37.9%)	0.030**
Eating faster than other people, n (%)		520 (38.2%)	614 (37.5%)	0.730
Having dinner within 2 h before going to sleep at least 3 times a week, n (%)		417 (30.6%)	442 (27.0%)	0.030**
Eating snacks after dinner at least 3 times a week, n (%)		439 (32.2%)	441 (26.9%)	0.002**
Skipping breakfast at least 3 times a week, n (%)		379 (27.8%)	405 (24.7%)	0.060
Insufficient sleep, n (%)		821 (60.3%)	909 (55.5%)	0.010**
Rome III criteria question items				
Improvement with defecation, n (%)		964 (70.8%)	1163 (71.0%)	0.904
Onset associated with a change in frequency of stool, n (%)		789 (57.9%)	957 (58.4%)	0.795
Onset associated with a change in form or appearance of stool, n (%)		753 (55.3%)	961 (58.7%)	0.064
Straining during at least 25% of defecation, n (%)		989 (72.6%)	1050 (64.1%)	< 0.001**
Lumpy or hard stool at least 25% of defecations, n (%)		933 (68.5%)	959 (58.5%)	< 0.001**
Sensation of incomplete evacuation for at least 25% of defecation, n (%)		1040 (76.4%)	1018 (62.1%)	< 0.001**
Sensation of hardly any evacuation, n (%)		1119 (82.2%)	1172 (71.6%)	< 0.001**
Manual maneuvers to facilitate digital evacuation at least 25% of defecation, n (%)		95 (7.0%)	67 (4.1%)	0.001**
Manual maneuvers to facilitate support of the pelvic floor at least 25% of defecation, n (%)		222 (16.3%)	161 (9.8%)	< 0.001**
Loose stools are rarely present without the use of laxatives, n (%)		753 (55.3%)	352 (21.5%)	< 0.001**
Quality of life (SF-8)				
GH^†^ (mean ± SD)		45.5 ± 8.1	46.8 ± 7.4	< 0.001**
PF^‡^ (mean ± SD)		46.2 ± 9.8	47.5 ± 8.3	< 0.001**
RP^§^ (mean ± SD)		47.6 ± 8.2	48.3 ± 7.6	0.003**
BP^¶^ (mean ± SD)		47.9 ± 8.9	48.5 ± 8.3	0.041**
VT^††^ (mean ± SD)		46.0 ± 8.2	47.2 ± 7.5	< 0.001**
SF^‡‡^ (mean ± SD)		45.4 ± 10.8	46.6 ± 9.4	< 0.001**
MH^§§^ (mean ± SD)		46.6 ± 8.8	48.0 ± 7.7	< 0.001**
RE^¶¶^ (mean ± SD)		43.0 ± 11.1	43.5 ± 10.6	0.169
PCS^†††^ (mean ± SD)		46.5 ± 7.8	47.3 ± 7.1	0.003**
MCS^‡‡‡^ (mean ± SD)		44.0 ± 9.7	45.1 ± 8.7	0.001**

*The level of significance is set at *P* < 0.05

**Significant difference between groups

^†^*GH* general health, ^‡^*PF* physical functioning, ^§^*RP* role physical, ^¶^*BP* body pain, ^††^*VT* vitality, ^‡‡^*SF* social functioning, ^§§^*MH* mental health, ^¶¶^*RE* role emotional, ^†††^*PCS* physical component summary, ^‡‡‡^*MCS* mental component summary

Compared to those in the strong awareness group, a higher proportion of subjects in the weak awareness group indicated that they were retired or unemployed (17.0% vs. 14.0%; *P* = 0.030). Conversely, there were significantly more homemakers in the strong awareness group (17.5% vs. 13.9%; *P* = 0.006). Additionally, the strong awareness group had a significantly higher level of education, with a bachelor’s degree or over (52.3% vs. 39.7%; *P* = 0.020).

A past medical history of anemia (19.3% vs. 14.7%; *P* < 0.001) and cardiovascular disease (3.5% vs. 2.1%; *P* = 0.030) were significantly more frequent in the strong awareness group, whereas a history of hypertension (14.7% vs. 11.8%; *P* = 0.020) was more frequent in subjects with weak awareness of constipation (Table 3).

#### Lifestyle

The evaluation of lifestyle using the JHPI survey showed that subjects with weak awareness of constipation more frequently indicated that they “had exercised aerobically for ≥30 minutes twice a week for at least 1 year” and “walk faster than other people of the same age” than those with strong awareness of constipation. Subjects with a strong awareness of constipation responded more frequently that they have “dinner within 2 h before going to sleep ≥ 3 times a week”, a “snack after dinner ≥ 3 times a week”, “skip breakfast ≥ 3 times a week,” and do not “get enough sleep”. This implies that subjects with an unhealthy lifestyle had a strong awareness of constipation.

#### Quality of life

Evaluation of the QOL of participants using the SF-8 questionnaire revealed that subjects with a weak awareness of constipation had a significantly higher MCS (45.1 ± 8.7, vs. 44.0 ± 9.7; *P* < 0.001) and physical component summary (PCS) than those with a strong awareness of constipation (Table 3).

#### Clinical symptoms

Comparison between the two groups based on the Rome III criteria revealed that the following conditions occurred significantly more frequently in subjects with strong awareness of constipation: straining, hard stool, sensation of incomplete evacuation, anorectal obstruction, manual maneuvering to facilitate evacuation, and rare bowel movements without the use of laxatives (Table 3).

Subjects with a strong awareness of constipation had a significantly higher percentage of stools corresponding to Bristol stool scale types 6 and 7, whereas those with weak awareness had types 4 (normal stool) and 5 (Fig. 2).

#### Significant background factors

Significant factors for strong awareness on univariate analysis were included in the multivariate logistic regression analysis, using a detection rate of ≤0.2%. Female sex, sensation of incomplete evacuation for at least 25% of instances of defecation, sensation of hard evacuation, and rare occurrence of loose stools without the use of laxatives were significant background factors related to a strong awareness of constipation. Conversely, onset associated with a change in frequency of stools, and Bristol stool type 4 were associated with weak awareness of constipation. (Table 4).

**Table 4:** Multivariate analysis of significant background factors for strong awareness of constipation

Items	univariate				multivariate			
	Odds	95% CI		***P*** value*	Odds	95% CI		***P*** value*
Age	1.002	0.996	1.007	0.558				
Sex (female), %	0.581	0.502	0.672	< 0.001**	0.729	0.614	0.867	< 0.001**
BMI	0.970	0.950	0.990	0.004**				
Jobs and education								
Office worker	0.986	0.854	1.140	0.853				
Retired or unemployed	0.726	0.588	0.895	0.003**				
Homemaker	1.323	1.085	1.612	0.006**				
Bachelor’s degree or over	0.730	0.631	0.844	< 0.001**	0.849	0.721	1.000	0.050**
Past history of disease								
Hypertension	0.794	0.646	0.976	0.029**	0.692	0.545	0.878	0.003**
Diabetes	1.083	0.812	1.444	0.589				
Dyslipidemia	0.994	0.810	1.219	0.952				
Anemia	1.141	0.985	1.321	0.078				
Rome III criteria question items								
Improvement with defecation	0.989	0.845	1.159	0.894				
Onset associated with a change in frequency of stool	0.980	0.847	1.134	0.784				
Onset associated with a change in form or appearance of stool	0.871	0.753	1.007	0.062	0.783	0.664	0.924	0.004**
Straining during at least 25% of defecation	1.485	1.270	1.736	< 0.001**				
Lumpy or hard stool at least 25% of defecations	1.450	1.324	1.790	< 0.001**				
Sensation of incomplete evacuation for at least 25% of defecation	1.967	1.676	2.308	< 0.001**	1.457	1.202	1.790	0.001**
Sensation of hard evacuation	1.831	1.536	2.182	< 0.001**	1.301	1.022	1.654	0.033**
Manual maneuvers to facilitate digital evacuation for at least 25% of defecation	1.759	1.276	2.435	< 0.001**				
Manual maneuvers to facilitate support of the pelvic floor for at least 25% of defecation	1.786	1.438	2.220	< 0.001**				
Loose stools rarely occurring without the use of laxatives	4.516	3.852	5.295	< 0.001**	4.070	3.439	4.817	< 0.001**
Bristol stool type 4	0.398	0.333	0.476	< 0.001**	0.549	0.450	0.671	< 0.001**
Source of laxative								
History of laxative use	2.887	2.483	3.358	< 0.001**				
By prescription	1.572	1.329	1.859	< 0.001**				
Pharmacy	1.375	1.189	1.590	< 0.001**				
Online	1.561	1.210	2.013	< 0.001**				
Acceptable laxative cost								
More than 1000 yen	2.083	1.762	2.482	< 0.001**				

*The level of significance is set at *P* < 0.05

**Significant difference between groups

## Discussion

The characteristic features of FC were evaluated in this study, based on responses from subjects included according to the Japanese population composition ratio. Those with organic diseases, and those using antidepressants or other medications for the treatment of thyroid diseases, diabetes mellitus, or hypertension, which can induce secondary constipation, were excluded. A study on the long-term prognosis of constipation, based on the population composition ratio, and excluding patients with cancer and comorbidities, has shown that constipation is associated with a high mortality rate [6]. However, the prognosis and prognostic factors for FC have not been elucidated. In the present study, FC was found predominantly in women, individuals with a low BMI, and the elderly (aged 60–69 years). In the context of age, FC is common in the elderly owing to various factors including inadequate exercise, inadequate dietary fiber intake, and imbalance of the autonomic nervous system. While all members of the FC group reported difficulties in having bowel movements without laxatives, 30% of those in the non-FC group also reported this symptom. Among the lifestyle factors, the only significant difference between groups was a history of previous weight gain of ≥10 kg in the non-FC subjects; thus, no association was found between lifestyle and FC. Interestingly, a strong awareness of constipation was considered a significant risk factor for FC (Table 1). Healthy lifestyle habits of exercising > 30 min twice a week and walking faster than other people of the same age were significantly less common in subjects with strong awareness of constipation. Additionally, the unhealthy habits of having late dinners (i.e. 2 h before sleep), getting snacks after dinner at least thrice a week, skipping breakfast at least thrice a week, and getting insufficient sleep were significantly more common in those with strong awareness. Evaluation of QOL using the SF-8^15^ revealed that subjects without FC had significantly lower scores for the vitality, social functioning, role emotional, and mental health subscales, whereas subjects with FC had significantly higher MCS. Irritable bowel syndrome (IBS-C) is known to have a strong negative impact on psychological status [16, 17], and the results of this study confirmed that FC is associated with a lower psychological impact than IBS-C, probably reflecting the poor QOL among those with IBS. The findings also suggested that a significantly higher percentage of subjects with FC were female and homemakers, and there were more office workers, retirees, and unemployed subjects in the non-FC group. Constipation is known to be prevalent in women of reproductive age [18]. Anatomically, Japanese women are believed to have longer colons [19]. Nevertheless, a comparison of the colorectal length between American and Japanese men and women aged over 50 years revealed no significant differences in colorectal length based on gender and nationality. Therefore, constipation in females or in specific ethnic groups cannot be attributed to a greater colorectal length [20]. In the present study, the male to female ratios among subjects in their 60s from the FC and non-FC groups were equal (data not shown); this suggests an association between FC and women of reproductive age.

Regarding the purchase of laxatives, a significantly higher percentage of subjects with FC used laxatives, purchased either in pharmacies and online; they considered ≥1000 to ≤5000 yen as an acceptable cost per month for the treatment of FC without a prescription. Widely available OTC laxatives often cause significant irritation of the gastrointestinal tract [21]; appropriate medical advice from a physician is therefore recommended for symptom relief in both IBS-C and FC. Some researchers have suggested the inclusion of IBS-C and FC within the same disease spectrum; although the relationship between gut microbiota and IBS-C has been investigated [21], further studies are needed to determine whether a similar relationship exists between FC and gut microbiota [16]. In an online survey conducted in the USA, the prevalence of chronic constipation was reported to be 5.5% according to the Rome III criteria, and it was found to be 2.1% based on medical consultations in Japan [13, 16]. According to the 2017 Comprehensive Survey of Living Conditions in Japan [22], constipation is more prevalent in women than in men. However, the number of men with constipation increases at the age of > 70 years, with approximately equal occurrence at the age of ≥80 years.

Awareness of constipation appears to stem from the subjective experience of its symptoms and from objective indices, such as stool frequency and consistency noted by a physician. Symptoms such as straining, sensation of incomplete evacuation, and sensation of anorectal obstruction are included in the Rome IV criteria [1] and Clinical Guidelines for Chronic Constipation [4]. Therefore, it is important that the physician appropriately considers these symptoms during clinical consultation. The association of a dysuria-type constipation suggests an imbalance in the defecation muscles or rectal pooling. Detailed evaluation of the chief complaint through careful history-taking may therefore lead to a correct diagnosis of constipation.

Those having a strong awareness of constipation complained of incomplete defecation and hard stools (*n* = 1119, 82.2%) more frequently. Infrequent reports of normal stools indicate a higher prevalence of incomplete bowel movements, probably explaining the subjective feeling of not having a good bowel movement without the aid of laxatives and the increase in laxative use. This study shows that weak constipation awareness and good lifestyle are related. This was evident from the fact that those with good health habits (i.e. walked faster than people of the same age, did not eat midnight snacks, did not skip breakfast, and had sufficient sleep) were less likely to have constipation and were consequently less aware of the condition. The fact that the number of people who were weakly aware of constipation (including defecation being rare without laxative use) was significantly lower indicated that lifestyle-related habits relieve the symptoms of constipation (Table 4). After considering the questionnaire item of loose stools being rare without laxatives and eliminating secondary constipation, only 262 of 3000 patients in this study were diagnosed with FC. However, 55.3 to 82.2% of participants responding to questions regarding the Rome III criteria (other than the two manual questions) indicated that these items were applicable, demonstrating their usefulness in diagnosing chronic constipation (Table 3).

The present survey was performed over the Internet, where the risk of impersonation cannot be eliminated completely. However, the respondents, who were registered panelists and whose identities can be confirmed by the survey company, were considered reliable. The population surveyed comprised 3000 participants, who were randomly extracted based on the population composition ratio by prefecture, sex, and age, thus reflecting the demographic profile in Japan. Nevertheless, as the upper age limit was set at 70 years in consideration of internet use among the elderly, further studies need to be conducted for generalization of these results among Japanese individuals aged 70 years and over. Despite these limitations, internet surveys are highly useful in performing cross-sectional studies on the actual status of certain conditions or issues; they offer the advantage of obtaining answers directly from participants, without any intervention or bias from healthcare personnel. This study involved a short data collection period for a large population size, with the selected sample reflecting the population composition ratio. The findings therefore provide valuable insights into the epidemiological factors of FC in Japan. The study was novel in that it evaluated the background factors involved in the epidemiology of functional constipation in the Japanese population. Although various objective indicators have been employed as diagnostic criteria for constipation in numerous studies, reports on background and lifestyle-related factors that cause the subjective state of constipation awareness are lacking.

## Conclusions

The prevalence of FC as a form of chronic constipation was low in participants who were representative of the Japanese population. FC was found more commonly in thin individuals, women, and those with a strong awareness of the condition. The QOL was only slightly reduced, indicating that patients with FC do not typically regard their constipation symptoms as serious health concerns. These patients tend to have an irregular lifestyle, purchase laxatives in pharmacies, and are willing to pay a higher amount for OTC remedies, suggesting a predisposition to self-treatment of their condition. Appropriate treatment for FC should be provided based on an understanding of these characteristic features, while primarily considering the symptoms and lifestyle in individual cases. In order to ensure that appropriate management of constipation is widely available, patients should be well-informed of their condition and the corresponding plan of intervention. Further large-scale prospective studies in diverse cohorts are needed to validate our findings.

## Data Availability

The datasets used and/or analyzed during the current study are available from the institutional review board (if permitted and on reasonable request) to use in a legitimate manner.

## Abbreviations

FC: functional constipation
IBS: irritable bowel syndrome
OTC: over-the-counter
QOL: quality of life
JHPI: Japanese Health Practice Index
SF-8 questionnaire: Short Form-8 questionnaire
GH: general health
PF: physical functioning
RF: role functioning
BP: bodily pain
VT: vitality
SF: social functioning
MH: mental health
RE: role emotional
PCS: physical component summary
MCS: mental component summary
BMI: body mass index
